# Feasible kidney donation with living marginal donors, including diabetes mellitus

**DOI:** 10.1002/iid3.470

**Published:** 2021-06-08

**Authors:** Kasumi Yoshinaga, Motoo Araki, Koichiro Wada, Takanori Sekito, Shogo Watari, Yuki Maruyama, Yosuke Mitsui, Takuya Sadahira, Risa Kubota, Shingo Nishimura, Kohei Edamura, Yasuyuki Kobayashi, Katsuyuki Tanabe, Hidemi Takeuchi, Masashi Kitagawa, Shinji Kitamura, Jun Wada, Masami Watanabe, Toyohiko Watanabe, Yasutomo Nasu

**Affiliations:** ^1^ Department of Urology, Graduate School of Medicine, Dentistry and Pharmaceutical Science Okayama University Okayama Japan; ^2^ Department of Nephrology, Rheumatology, Endocrinology and Metabolism, Graduate School of Medicine, Dentistry and Pharmaceutical Science Okayama University Okayama Japan

**Keywords:** diabetes mellitus, kidney function, kidney transplantation, marginal donor

## Abstract

**Objectives:**

To compare the donor outcomes of living donor kidney transplantation between standard donors (SDs) and marginal donors (MDs) including diabetic patients (MD + DM).

**Methods:**

MDs were defined according to Japanese guideline criteria: (a) age >70‐years, (b) blood pressure ≤130/80 mmHg on hypertension medicine, (c) body mass index >25 to ≤32 kg/m^2^, (d) 24‐h creatinine clearance ≥70 to <80 ml/min/1.73 m^2^, and (e) hemoglobin A1c > 6.2 or ≤6.5 with oral diabetic medicine. Fifty‐three of 114 donors were MDs. We compared donor kidney functions until 60 months postoperatively.

**Results:**

No kidney function parameters were different between SDs and MDs. When comparing SD and MD + DM, MD + DM had a lower postoperative eGFR (48 vs. 41 (1 (month), *p* = .02), 49 vs. 40 (12, *p* < .01), 48 vs. 42 (24, *p* = .04), 47 vs. 38 (36, *p* = .01)) and the percentage of residual eGFR (SD vs. MD + DM: 63 vs. 57 (1 (month), *p* < .01), 63 vs. 57 (2, *p* < .01), 64 vs. 56 (12, *p* < .01), 63 vs. 57 (24, *p* < .01), 63 vs. 52 (36, *p* = .02)). However, when MD with a single risk factor of DM was compared to SD, the difference disappeared. Nine out of 12 (75%) MD + DM had ≥2 risk factors.

**Conclusions:**

Although long‐term observation of donor kidney function is necessary, careful MD + DM selection had the potential to expand the donor pool.

## Graphical abstract

This study demonstrates the safety and potential of kidney donations for marginal donors. Careful Mmarginal donors with diabetes mellitus selection has the potential to expand the donor pool.

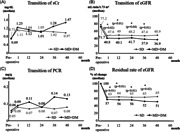

**Abbreviations**
ACR: urine albumin‐creatinine ratio
BMI: body mass index
BP: blood pressure
DM: diabetes mellitus
eGFR: estimated glomerular filtration rate
ESRD: end‐stage renal disease
HbA1c: hemoglobin A1c
HTN: hypertension
IQR: interquartile range
MD: marginal donor
MD + DM: marginal donor with diabetes mellitus
MMF: mycophenolate mofetil
PCR: urine protein‐creatinine ratio
sCr: serum creatinine
SD: standard donor
Tac‐ER: extended release tacrolimus

## 1 INTRODUCTION

Kidney transplantation is the most useful treatment for patients with end‐stage renal disease (ESRD). The prevalence of ESRD in Japan was the second highest in the world in 2016, with 2599 cases per million of the general population.^1^ Despite the increasing number of ESRD patients, the donor shortage has been a serious problem worldwide, particularly in Japan, with only 1865 (0.5%) kidney transplantations compared with approximately 340,000 dialysis patients in 2018.^2^, ^3^ Furthermore, most transplants were from living‐donors (1683 cases), with only 55 coming from non‐heart‐beating donors and 127 coming from heart‐beating donors.^3^

In 2014, the original marginal donor (MD) criteria were established in Japan with reference to the standards for live‐donor kidney transplants put forth by the Amsterdam Forum.^4^, ^5^ To address the gap between the number of patients awaiting kidney transplants and the number of kidney donors, the number of MD with borderline kidney conditions and potential to be kidney donors is increasing following detailed preoperative testing. MDs may now include people of older age, people with hypertension (HTN), and those who are obese, have mild kidney dysfunction, or diabetes mellitus (DM). Other factors also playing a role in the increase in MDs are an aging population, an increase in the number of people with lifestyle‐related diseases, and an increase in the number of living‐donor kidney transplantations among couples or parents and children. One of the most notable Japanese criteria for kidney transplants is that donors with DM are approved if their blood glucose is well‐controlled without the need for insulin. This exception is not currently permitted in most other countries because the presence of DM in donor is not allowed in the Amsterdam Forum.^4^, ^5^ However, the adequacy of MD with DM (MD + DM) is currently unknown, since the treatment for DM continues to evolve.

A total of 19.6% of kidney donors in Japan had a history of HTN and 4.1% of kidney donors had a history of diabetes in 2018 and those rates are incrising.^3^, ^6^ In the same year, 45.2% of living donors with HTN were over 60 years old and 15.4% with diabetes were over 70 years old.^3^ In the United States, 5.2% of kidney donors were older than 65 years, indicating that living kidney donors were generally older in Japan.^7^

It is essential that a donated kidney is functionally adequate, and also that the selected MD's kidney function and health condition is not affected after the donation in the long‐term. The definition of marginal criteria differs by reports; however, several reports have indicated the usefulness and safety of kidney donation from MDs.^8^, ^9^ In contrast, there are reports that kidney donations from MD may result in worse long‐term outcomes than from standard donor (SD) for both the recipients and donors, underscoring the need for careful selection.^10^, ^11^, ^12^

This study investigated the adequacy of the current selection criteria for MDs based on the Japanese guidelines, including those with DM, and assessed the changes in renal function of donors after donation.

## 2 MATERIALS AND METHODS

### 2.1 Patients

In this study, we retrospectively evaluated 114 patients who underwent donor nephrectomy at our institution between May 2009 and April 2020. One hundred ten cases (96%) of donor nephrectomies were performed laparoscopically. We compared the backgrounds of donors at the time of transplantations including age, gender, marital status and each marginal factor. Based on the guidelines for living‐donor renal transplantations in Japan, we defined donors as marginal when at least one of the following conditions was met: (a) age >70 years, (b) blood pressure (BP) ≤ 130/80 mmHg with taking antihypertensive drugs, (c) 25 < body mass index (BMI) ≤ 32 kg/m^2^, (d) 70 ≤ creatinine clearance <80 ml/min/1.73 m^2^ per 24‐h urine collection, (e) 6.2 < hemoglobin A1c (HbA1c) ≤ 6.5 without oral medication for diabetes or ≤6.5 with oral medication for diabetes.^4^ All cases met the donor criteria with a urine albumin‐creatinine ratio (ACR) of <30 mg/g.

Our retrospective study complied with the standards of the Declaration of Helsinki and current ethical guidelines, and was approved by the Okayama University Institutional Review Board (Registration No.: 1908‐026). We obtained written informed consent from all patients for the use of their clinical records.

### 2.2 Induction protocol

Triple therapy, including extended‐release tacrolimus (Tac‐ER), mycophenolate mofetil (MMF), and prednisolone plus basiliximab, was the induction therapy. Basiliximab was administered on the day of the transplantation and 4 days later. ABO major mismatch, ABO minor mismatch, preformed DSA, and focal segmental glomerulosclerosis cases received a 200 mg/body dose of rituximab 1–2 weeks before the date of renal transplantation in addition to induction therapy. Tac‐ER, MMF, and prednisolone were also used as maintenance therapy.

### 2.3 Renal function

Donors kidney function transitions were compared. To evaluate kidney function, serum creatinine (sCr), estimated glomerular filtration rate (eGFR), and urine protein‐creatinine ratio (PCR) were measured preoperatively and postoperatively at 1, 12, 24, 36, 48, and 60 months. The percentage of residual eGFR from baseline was also calculated. The baseline eGFR was defined as preoperative one. ACR was also assessed preoperatively and at discharge. MD + DM were subsequently compared with SDs in the same way.

### 2.4 Statistical analysis

All statistical analyses were performed using EZR version 1.41 (Saitama Medical Center, Jichi Medical University, Saitama, Japan), a graphical user interface for R.^13^ Donor's characteristics and kidney function data were compared between patients with SD and MD using Fisher's exact test for categorical variables, and Mann–Whitney *U* test was used for continuous variables. *p* < .05 were considered significantly different.

## 3 RESULTS

**Figure 1:**
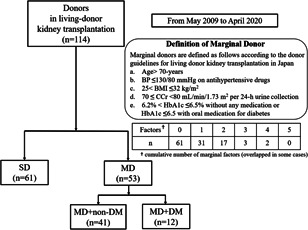
Study flow chart. The flow chart shows the derivation of the two groups. BMI, body mass index; BP, blood pressure; CCr, creatinine clearance; DM‐MD, marginal donor with diabetes mellitus; HbA1c, hemoglobin A1c; MD, marginal donor; SD, standard donor

There were 61 SDs and 53 MDs. As shown in Figure 1, 20 donors met the MD criteria despite having multiple risk factors. Table 1 shows the characteristics of donors and Table 2 lists the risk breakdown for MDs. Only median age was significantly different between SD and MD (57 vs. 64 years, respectively; *p* < .01), but gender and marital status were similar between groups. Median age of MD + DM was 71 (interquartile range [IQR]: 68–74) and that of MD without DM was 62 (IQR: 56–66). HTN and high BMI were the most common risk factor among MD (*n* = 25) and suboptimal kidney function was the least common risk factor (*n* = 5).

**Table 1:** Donor's characteristics

Total cases, *n* (%)	Total	*SD*	MD	*p* value
	114 (100)	61 (54)	53 (46)	
*Donor's background*				
Age, median (IQR)	60 (53–66)	57 (51–62)	64 (57–71)	<.001
Sex (male), *n* (%)	49 (43)	30 (49)	19 (36)	.186
marital, *n* (%)	45 (39)	21 (34)	24 (45)	.255
*Marginal factor*				
Age >70‐year‐old, *n* (%)	15 (13)	0 (0)	15 (28)	<.001
BP ≤130/80 mmHg with taking antihypertensive drugs, *n* (%)	25 (22)	0 (0)	25 (47)	<.001
25 < BMI ≤ 32, *n* (%)	25 (22)	0 (0)	25 (47)	<.001
70** ≤** CCr < 80 ml/min/1.73m^2^ in 24‐h urine collection test, *n* (%)	5 (4)	0 (0)	5 (9)	.020
6.2 < HbA1c ≤ 6.5 without any medication or HbA1c ≤ 6.5 with oral medication for diabetes, *n* (%)	12 (11)	0 (0)	12 (23)	<.001

Abbreviations: BP, blood pressure; BMI, body mass index; CCr, creatinine clearance; HbA1c, hemoglobin A1c; IQR, interquartile range; MD, marginal donor; SD, standard donor.

**Table 2:** The risk breakdown for marginal donors

Marginal factors			
Number	Risk	Number of MDs (%)	
1	Age^a^	4 (8)	31 (59)
	HTN^b^	9 (18)	
	BMI^c^	13 (25)	
	CCr^d^	2 (4)	
	DM^e^	3 (6)	
2	Age + HT	2 (4)	17 (32)
	Age + BMI	1 (2)	
	Age + DM	4 (8)	
	HT + BMI	7 (13)	
	HT + CCr	2 (4)	
	BMI + DM	1 (2)	
3	Age + HT + BMI	1 (2)	3 (6)
	Age + HT + DM	1 (2)	
	HT + CCr + DM	1 (2)	
4	Age + HT + BMI + DM	2 (4)	2 (4)

Abbreviations: BMI, body mass index; CCr, creatinine clearance; DM, diabetes mellitus; HTN, hypertension.

^a^ Age > 70‐year‐old.

^b^ Blood pressure ≤130/80 mmHg with taking antihypertensive drugs.

^c^ 25 < BMI ≤ 32.

^d^ 70 ≤ CCr < 80 ml/min/1.73m^2^ in 24‐h urine collection test.

^e^ 6.2 < hemoglobin A1c ≤ 6.5 without any medication or hemoglobin A1c ≤ 6.5 with oral medication for diabetes.

Percentages does not add up to 100% due to rounding.

**Figure 2:**
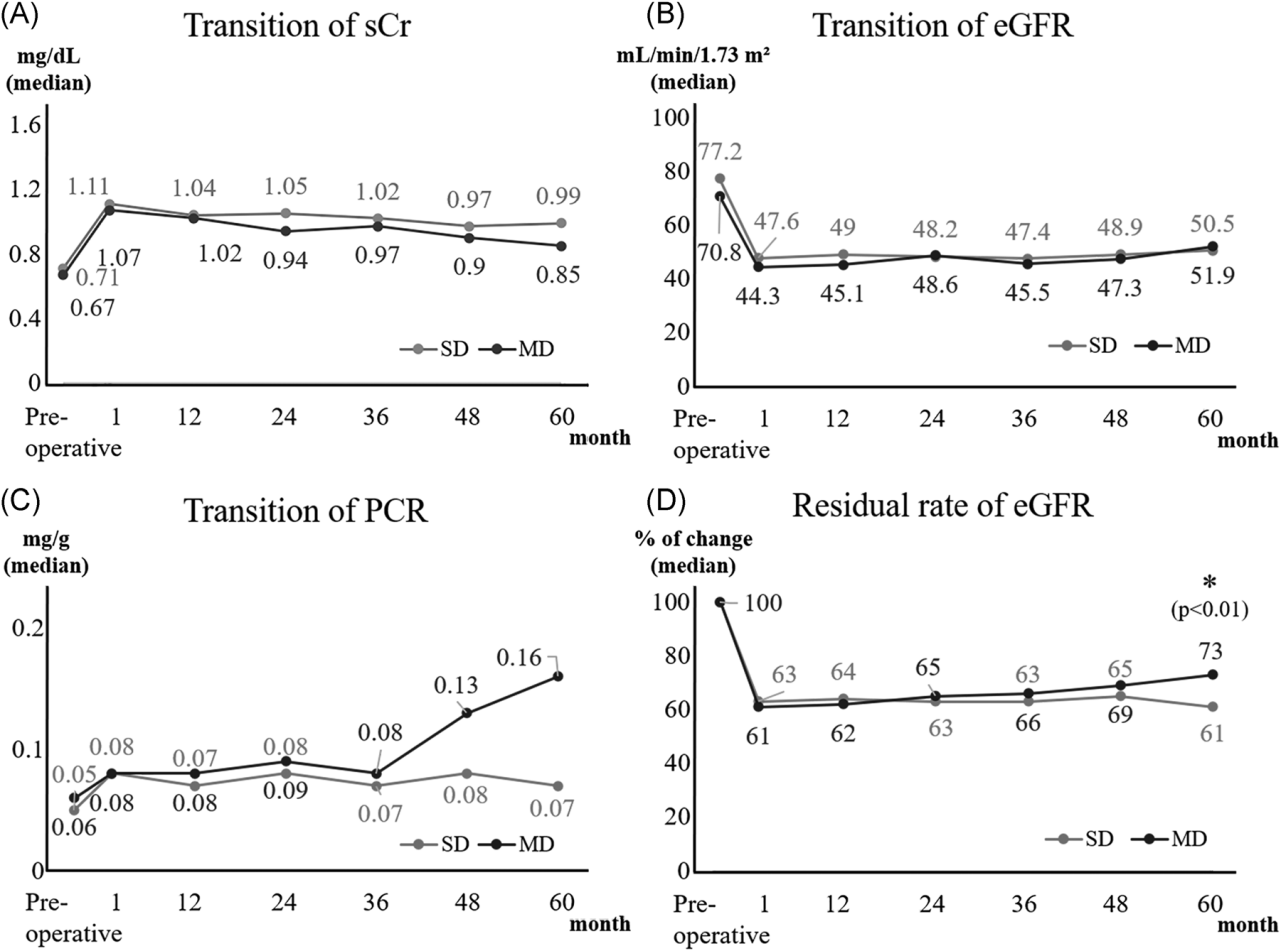
Kidney function transition of donors over 60 months in SD and MD groups. All kidney functions were similar between groups, except for the residual rate of eGFR at 60 months postoperatively (SD vs. DM; 61 vs. 73%, *p* < .01). eGFR, estimated glomerular filtration rate, MD, marginal donor; PCR, urine protein‐creatinine ratio; sCr, serum creatinine; SD, standard donor

Preoperatively, and up to 60 months postoperatively, donor kidney function did not differ significantly between the SD and MD groups (Figure 2). In that order, ACR preoperatively and at discharge, respectively, in donors was 6 versus 7 (*p* = .16) and 8 versus 9 (*p* = .55). All donors had a good postoperative course, with no cases of dialysis induction or death.

**Figure 3:**
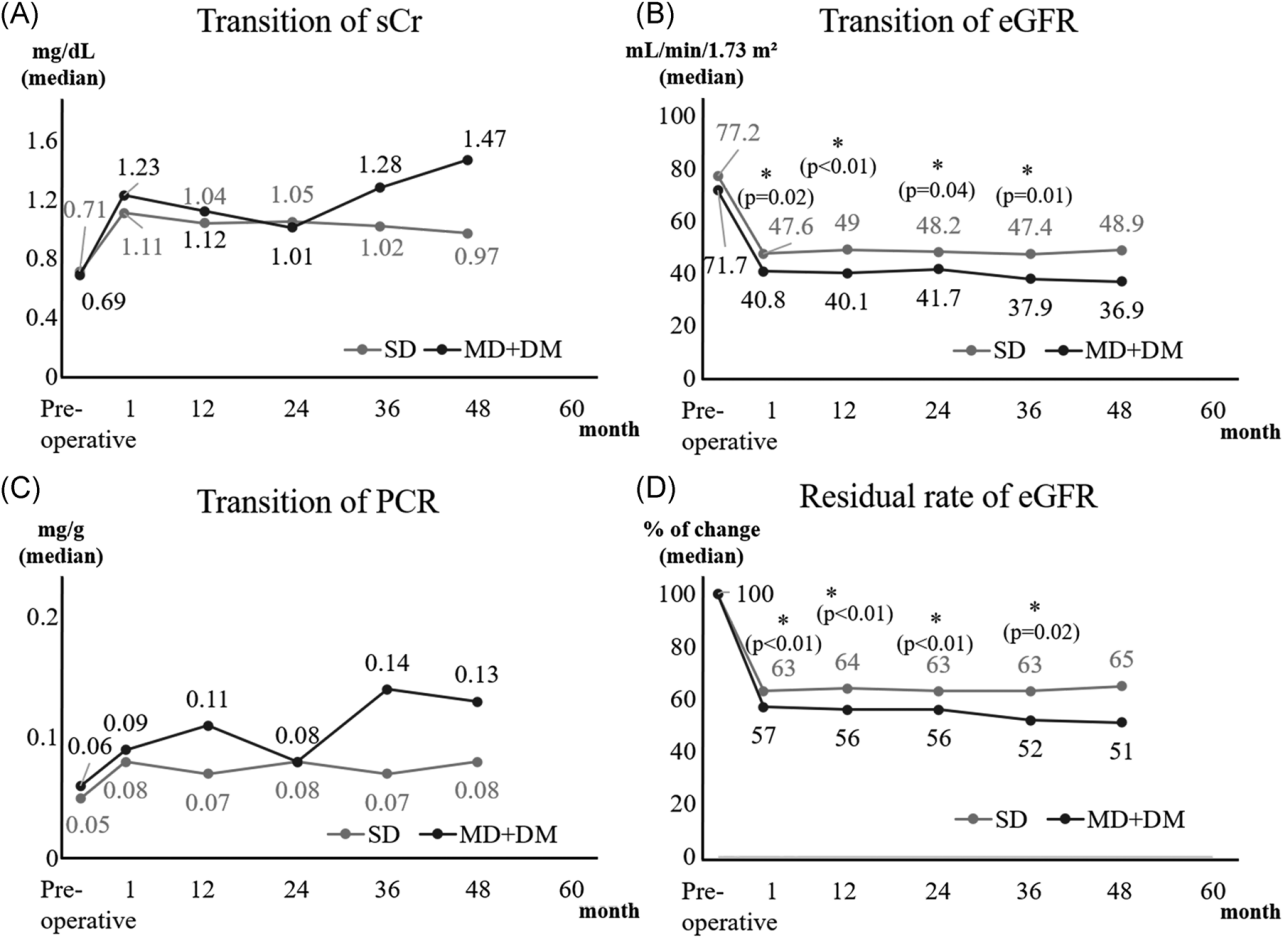
Kidney function transition of donors over 48 months in SD and DM‐MD. A significant difference in median parameter values was observed at 1 month (48 vs. 41 ml/min/1.73 m^2^, *p* = .02), 12 months (49 vs. 40 ml/min/1.73 m^2^, *p* = .01), 24 months (48 vs. 42 ml/min/1.73 m^2^, *p* = .04), and 36 months (47 vs. 38 ml/min/1.73 m^2^, *p* = .01) during the transition of eGFR. The residual rate of eGFR was lower in the DM‐MD versus the SD group. Other kidney functions were similar between groups. eGFR, estimated glomerular filtration rate; DM‐MD, marginal donor with diabetes mellitus; PCR, urine protein‐creatinine ratio; sCr, serum creatinine; SD, standard donor

**Figure 4:**
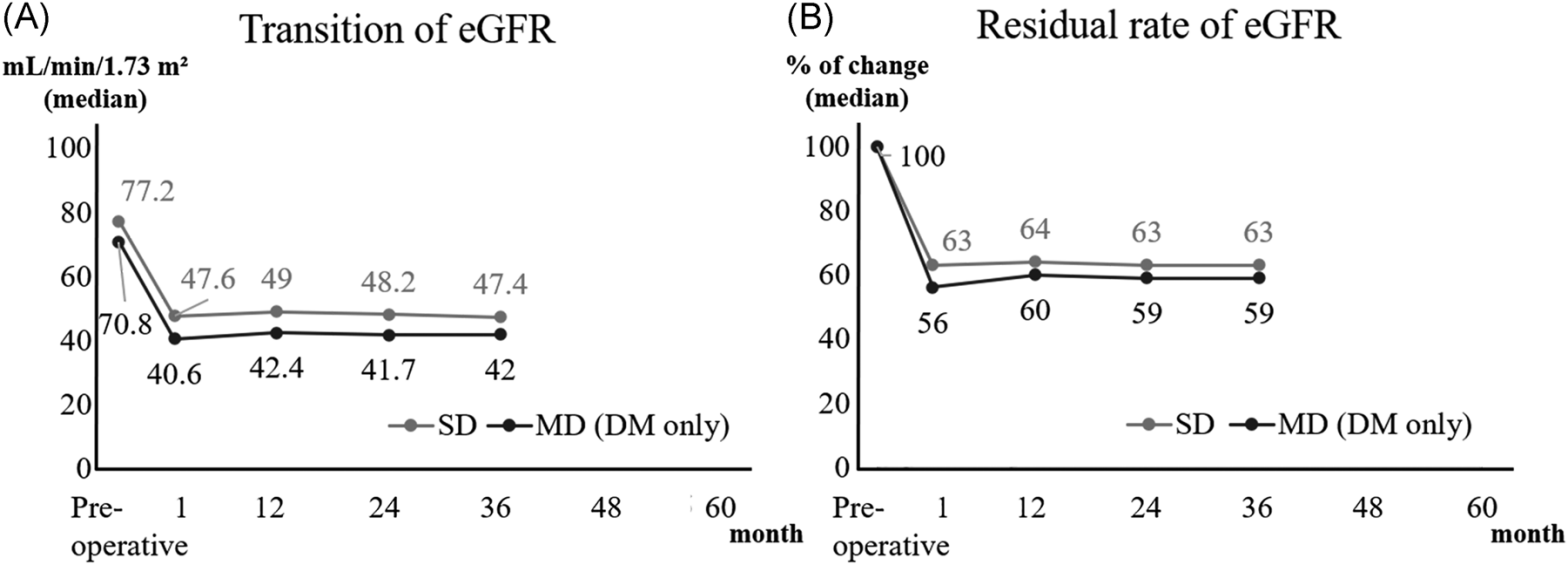
eGFR transition and the residual rate between the SD group and the MD group with the single risk factor of DM. DM, diabetes mellitus; eGFR, estimated glomerular filtration rate; SD, standard donor

The comparison between SD and MD + DM in donors could be analyzed for 48 months. Postoperative eGFR was lower in MD + DM, and residual rate of eGFR from baseline in MD + DM was significantly higher at any point compared with those in SDs. No significant differences between SDs and MD + DM were found in sCr, PCR (Figure 3) and ACR (preoperatively: 6 vs. 8, *p* = .04; at discharge: 8 vs. 9, *p* = .99). However, when compared to MD + DM without multiple risk factors for 36 months, the difference disappeared. Nine out of 12 (75%) MD + DM had ≥2 risk factors per the MD criteria (Figure 4).

## 4 DISCUSSION

The data from our study suggested that donor kidney function did not differ significantly between the SD and MD groups. However, we should carefully follow MD + DM with multiple risk factors since they had a lower kidney function compared with SD.

Previous reports comparing eGFR between SD and MD groups showed that MDs had a lower eGFR preoperatively and postoperatively; however, no significant differences in the rate of residual of eGFR were reported.^14^, ^15^, ^16^ Dols et al.^15^ showed no postoperative differences in the decline of eGFR and in proteinuria defined as ≥3.0 g/L between donors aged <60 and ≥60 years. The Kidney Disease: Improving Global Outcomes guidelines revised the classification system for chronic kidney disease to include albuminuria.^17^ The earliest clinical course of diabetic nephropathy is recognized by measuring microalbuminuria. Of note, ACR is not an acceptable measurement in Japan national health insurance except when diabetes is diagnosed. PCR is measured instead because proteinuria is also reported to predict the risk of ESRD.^18^ We were able to measure ACR preoperatively, at discharge and at 12 months postoperatively and found no significant differences between groups. Despite the limitation of a short observation period of 5 years, none of the donors in this study experienced ESRD or died after donating a kidney. In summary, our selection of MDs was exceedingly careful and is reflected in the results as no significant difference in the preoperative eGFR of MDs when compared with SDs.

We also compared the changes in renal function between SD and MD + DM. Our results showed that the postoperative eGFR was significantly lower in MD + DM. However, the differences disappeared comparing with the MD + DM without multiple risk factors. Our results indicated that a kidney transplantation from MD + DM was possible. It will help expand the donor pool. However, one should be careful to use MD + DM with multiple risk factors especially when they were young. The average age of MD + DM was 71 years in our study.

Systolic BP > 140/90 mmHg is important cardiovascular risk for both the recipient and donors during and after kidney transplantation.^11^, ^19^ Kidney donation is permitted in the event of mild‐to‐moderate HTN that is controlled with one or a combination of two antihypertensive drugs providing significant end‐organ damage has been excluded.^10^ Bozkurt et al.^11^ reported that there is no definitive evidence to suggest a direct effect of high BP on graft rejection after renal transplantation. At our institution, 20.2% of donors had controlled HTN preoperatively. Of the SDs, 12 had systolic BP > 140 postoperatively, and 5 of them started receiving one or more antihypertensive medications. The time to medication ranged from 1 to 62 months postoperatively. The other 7 have been treated with lifestyle changes including salt reduction or weight reduction.

Obesity is one of the significant risks affecting long‐term outcomes after kidney donation. BMI > 32 kg/m^2^ is a contraindication for kidney donation according to the Japanese criteria.^4^ Two obesity‐related disorders can cause kidney disease: (1) glucose intolerance and HTN associated with obesity, and (2) obesity‐related glomerulopathy, caused by obesity itself.^20^ No consensus has been reached as to whether obesity is a risk for the decline of kidney function in donors.^21^, ^22^ There is a report indicating that the incidence of HTN and other cardiovascular diseases in obese donors was higher than in nonobese donors.^23^ A donor nephrectomy is a more difficult procedure to perform in a living donor who is obese. At our institution, we have never performed donor nephrectomy until a donor's BMI is <28.3 kg/m^2^ after dieting.

All donors have visited the hospital at 1 month, 2 months, and then every year after kidney transplantation to check their health status, including HTN, obesity, decreased kidney function and DM. When the results of blood and urine examination and vital checks indicate the need for intervention, we have actively referred them to medical specialists and provided nutritional counseling. Our strict MD selection and careful postoperative follow‐up has contributed to no donor on dialysis after kidney transplantation.

All donors have visited the hospital at 1 month, 2 months, and then every year after kidney transplantation to check their health status, including HTN, obesity, decreased kidney function and DM. When the results of blood sampling and vital checks indicate the need for intervention, we have actively referred them to medical specialists and provided nutritional counseling. These severe MD selections and careful postoperative follow‐up have resulted in no donor being placed on dialysis intervention after kidney transplantation.

Comparing with the fact that MDs were 23%–29% in previous Japanese reports,^14^, ^16^ our MD rate is as high as 46%. MD's postoperative kidney function was not different from those of SD during the first 5 years. Our data suggested that MD including MD + DM is acceptable in carefully selected population. Especially, we selected MD + DM very carefully. We avoided young donor if a donor has DM. Median age of MD + DM was 71 (IQR: 68–74) and that of MD without DM was 62 (IQR: 56–66). We think that MD + DM is acceptable if donor is relatively aged. We live in an aging society and lifestyle‐related diseases increase. Our strategy will help expand the donor pool.

This study has some limitations. First, this was a small, retrospective study performed at a single center and thus has the potential for selection bias. Second, our observation period is only 5 years. Third, Japan has as high as 90% of living‐donor kidney transplantations, whereas in most countries kidneys are acquired from deceased donors.^3^ The recipient's primary disease and the marginal risk factors are disproportionate among countries. Although it is necessary to further verify the validity of our results, this study is significant in terms of expanding the donor pool. Third, we used sCr and eGFR as biomarkers of kidney function. Other data, such as serum cystatin C, should be examined in the future for the accurate evaluation of kidney function.^24^ Fourth, there is the lack of the information about the graft function outcomes in the recipients. Finally, we also think it is meaningful to compare the pathological changes in 0‐time biopsy between the SD and MD group, such as arteriosclerosis, arteriolosclerosis, interstitial fibrosis and tubular atrophy, glomerulosclerosis rate and glomerular abnormality. We are currently writing different papers on this topic.

Despite these limitations, this study demonstrates the safety and potential of kidney donations for MDs. The kidney function of MDs was not different when compared with SD during the first 5 years. Long‐term observation is needed in the future. Greater attention needs to be taken in the selection of MD + DM in terms of postoperative kidney function, particularly when MD candidates present with multiple risk factors.

## CONFLICT OF INTERESTS

The authors declare that there are no conflict of interests.

## AUTHOR CONTRIBUTIONS

Motoo Araki, Koichiro Wada, and Yasutomo Nasu were involved in study design and data interpretation. Kasumi Yoshinaga, Yuki Maruyama, Yosuke Mitsui, and Takuya Sadahira were involved in data analysis, drafted the manuscript and designed the figures. Takanori Sekito, Shogo Watari, Risa Kubota, Shingo Nishimura, Kohei Edamura, Yasuyuki Kobayashi, Katsuyuki Tanabe, Hidemi Takeuchi, Masashi Kitagawa, Shinji Kitamura, Jun Wada, Masami Watanabe, and Toyohiko Watanabe were were involved in critical revision of the manuscript for important intellectual content. All authors critically revised the report, commented on drafts of the manuscript, and approved the final report.

## Data Availability

The data that support the findings of this study are available on request from the corresponding author.
